# Differences between β-Ala and Gly-Gly in the design of amino acids-based hydrogels

**DOI:** 10.3762/bjoc.6.109

**Published:** 2010-10-11

**Authors:** Andreea Pasc, Firmin Obounou Akong, Sedat Cosgun, Christine Gérardin

**Affiliations:** 1 LERMAB - EA 4370, Nancy-Université, BP 70239, F-54506 Vandoeuvre-lès-Nancy, France; 2 Department of Chemistry, Fatih University, 34500 Buyukcekmece-Istanbul, Turkey

**Keywords:** amino acid, histidine, hydrogel, peptide-based surfactant, soft matter, supramolecular

## Abstract

Despite the continuous interest in organogels and hydrogels of low molecular weight gelators (LMWG), establishing the relationship between the molecular structure and the gelation mechanism is still a challenge. In this paper our interest focuses on the consequences of slight molecular modifications on the self-assembling behaviour of β-Ala vs Gly-Gly-based hydrogelators. Previously, in our group, amino acid based amphiphiles i.e. Gly-Gly-His-EO_2_-Alk, a trimodular amphiphile (containing three domains: H-bond donor and acceptor/hydrophilic/hydrophobic domain, respectively) were reported to act as hydrogelators and that the gelation properties were related to hydrogen bonding, hydrophobic interactions and π-π stacking. Herein, β-Ala-His-EO_2_-Alk was fully characterised by FT-IR, NMR, SAXS and SEM and the gelation mechanism is discussed. It appears that the number of amide groups determines the self-assembling behaviour into 1D or 2D/3D networks as a result of intimate interactions between gelator molecules.

## Introduction

Hydrogels continue to attract much interest due to their versatile applications in tissue engineering, biosensing, drug or gene delivery and water pollution control [1–7]. For some of these applications, i.e., drug controlled-release systems or bioseparation, hydrogels are required to respond to external stimuli such as temperature, pH and ions. They have been traditionally constructed with high molecular weight polymers, physically or chemically cross-linked, but in the recent past, their construction by low molecular weight (LMW) compounds has been explored. With respect to polymeric hydrogels, supramolecular gels of LMWGs (low molecular weight gelators) are assembled by non-covalent forces, such as electrostatic, hydrogen bonding, dipole–dipole, π–π stacking and hydrophobic/van der Waals interactions. Since the cross-links between fibres are non-covalent in nature, LMWHs exhibit thermoreversibility, rapid response to external stimuli (i.e., stirring, sonication). Among these LMWHs, amino acid-based amphiphiles have been received much attention in the recent past on account of their biocompatibility and eco-friendly nature.

Biologically inspired His-based surfactants are of particular interest not only for their antioxidant properties and their potential therapeutic effects, but also as building blocks for supramolecular architectures such as gels [8–9]. The structure of the surfactant is a major factor for their gelator properties. As reported in the literature [1], unless the molecules remain in solution, when a hot, homogeneous solution of the gelator, is cooled, the molecules start to condense and three outcomes are possible: (1) a highly ordered aggregation giving rise to crystals; (2) a random aggregation resulting in an amorphous precipitate, or (3) an aggregation process intermediate between these two, yielding a gel. The result seems to depend on the balance between hydrophilic and hydrophobic modules, as well as the number of H-donor and H-acceptor centers, or π–π stacking. Therefore the appropriate balance between all these factors must be determined.

As a part of the ongoing research carried out in our laboratory on the self-assembled systems of amino acids-based amphiphiles, we have recently shown that Gly-Gly-His and β-Ala-His-amphiphiles act as efficient hydrogelators [10]. The histidine moiety seems to play a key role in hydrogel formation, developing not only π-π stacking interactions but also by H-bonding; by replacing histidine by phenylalanine no hydrogel formation occurred (results to be published elsewere). His-based amphiphiles are surface active at low pH (< 7) and are able to complex Cu(II) ions to form metallosurfactants. Herein, the self-organisation within hydrogels (pH > 7) was investigated in order underline the role of slight variations of the molecular structure in supramolecular self-assembling, i.e., the number of H-bond donor and acceptor amide moieties (3 in β-Ala-His-amphiphile vs 4 Gly-Gly-His-amphiphile) and consequently, to obtain further insights on the gelation mechanism. The hydrogels were characterised by a number of techniques including, NMR, FT-IR, scanning electron microscopy, SAXS/WAXS.

## Results and Discussion

### Molecular design

**Figure 1:**
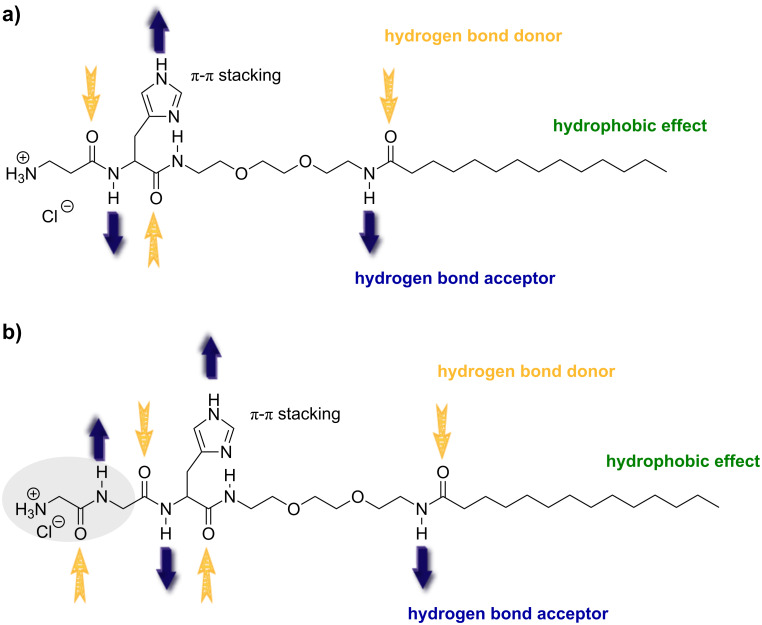
Molecular structures of β-Ala-His-EO_2_-C_14_ (a) and Gly-Gly-His-EO_2_-C_14_ (b).

A hydrogelator generally contains three functional domains: a hydrogen or electron donor and acceptor domain as the main organiser, a hydrophilic domain to adjust the solubility in water or in organic solvents, and a hydrophobic domain for van der Waals or hydrophobic interactions (Figure 1). They are able to entrap a large number of solvent molecules per one gelator molecule.

Herein the gelators were designed as AA-His-EO_2_-Alk, bearing i) an hydrophobic alkyl group, ii) a polar peptide group with complexing properties (AA = β-Ala or Gly–Gly) and iii) a flexible hydrophilic junction module, allowing for HLB control. Their synthesis is reported elsewere [11].

### Gelation behaviour of amphiphiles

In previous studies, we showed that amphiphilic compounds designed as AA-His-EO_2_-C*_n_*, with *n* = 12, 14 and AA = β-Ala or Gly-Gly, respectively, formed hydrogels [10–11]. Gly-Gly-His-EO_2_-C_14_ formed lamellar phases, the gels were transparent and the gel-to-sol transition temperatures were lower with respect to the β-Ala-derivative. In order to understand better the differences between the two compounds, NMR, X-ray and FT-IR measurements were performed on β-Ala-His-EO_2_-C_14_. The FT-IR measurements were made in order to evaluate the driving forces for the hydrogelation.

**Figure 2:**
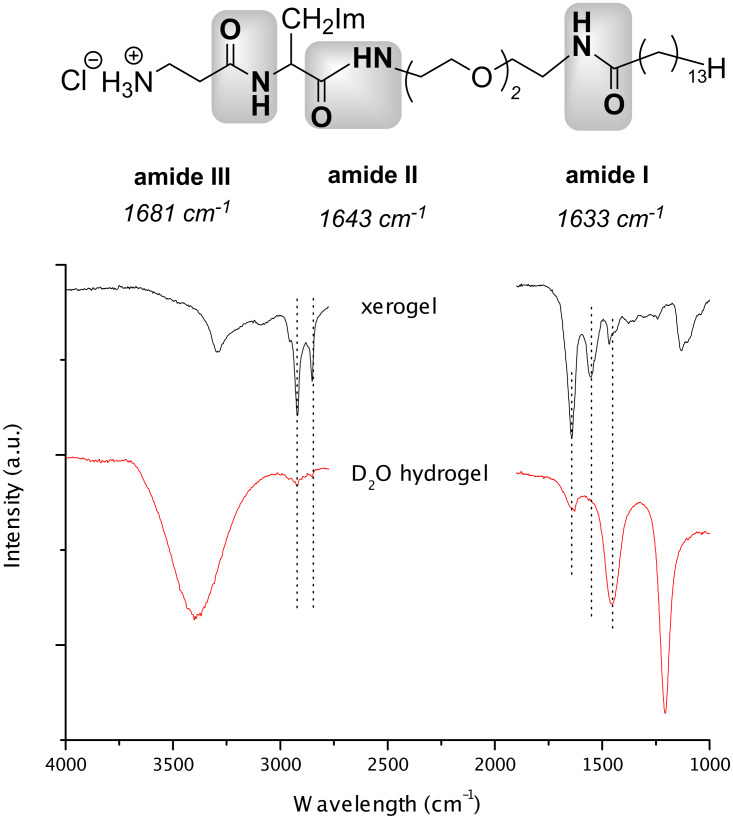
ATR spectra of β-Ala-His-EO_2_-C_14_ in xerogel and D_2_O hydrogel, respectively.

As expected ATR measurements (Figure 2) show the presence of H-bonding of the amides. The absorptions frequencies in the gel are always lower than in the free powder. The free individual IR frequencies of amide **I**-**III** were identified from the corresponding IR spectra of H_2_N-EO_2_-C_14_ (amide **I**, 1633 cm^−1^), His-EO_2_-C_14_ (amide **II**, 1643 cm^−1^) and β-Ala-His-EO_2_-C_14_ (amide **III**, 1681 cm^−1^), respectively. In the xerogel, only one absorption band was observed at 1644 cm^−1^, whereas in the D_2_O hydrogel a weak and broad absorption band appeared at 1635 cm^−1^, demonstrating the existence of H-bonded amide groups.

Furthermore, the increase in intensity of the methylene scissoring vibration δ(CH_2_) at 1454 cm^−1^ indicates the high conformational packing of alkyl chain.

The ^1^H NMR spectra of β-Ala-His-EO_2_-C_14_ D_2_O hydrogel (2% w/v) was carried out in order to both determine the sol-to-gel transition temperature and to see what group is implicated in the construction of the gel. In the gel state (below 30 °C) all gelator signals were poorly resolved; whereas above 35 °C they were more distinct and slightly right shifted.

**Figure 3:**
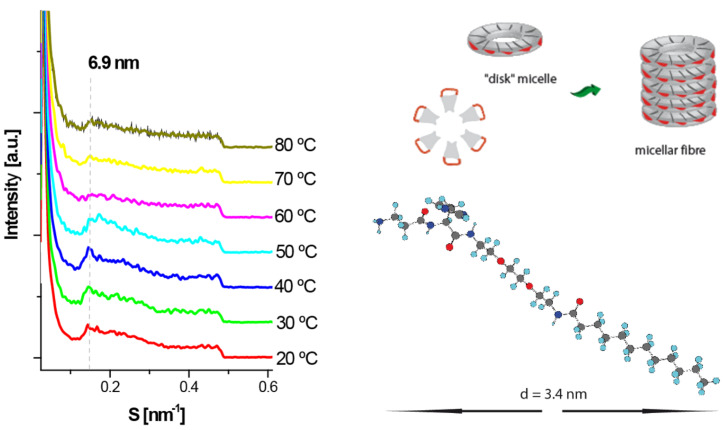
SAXS profile of concentrated gel of β-Ala-His-EO_2_-C_14_ at different temperatures (where S = 2π/q and q is the scattering vector).

**Figure 4:**
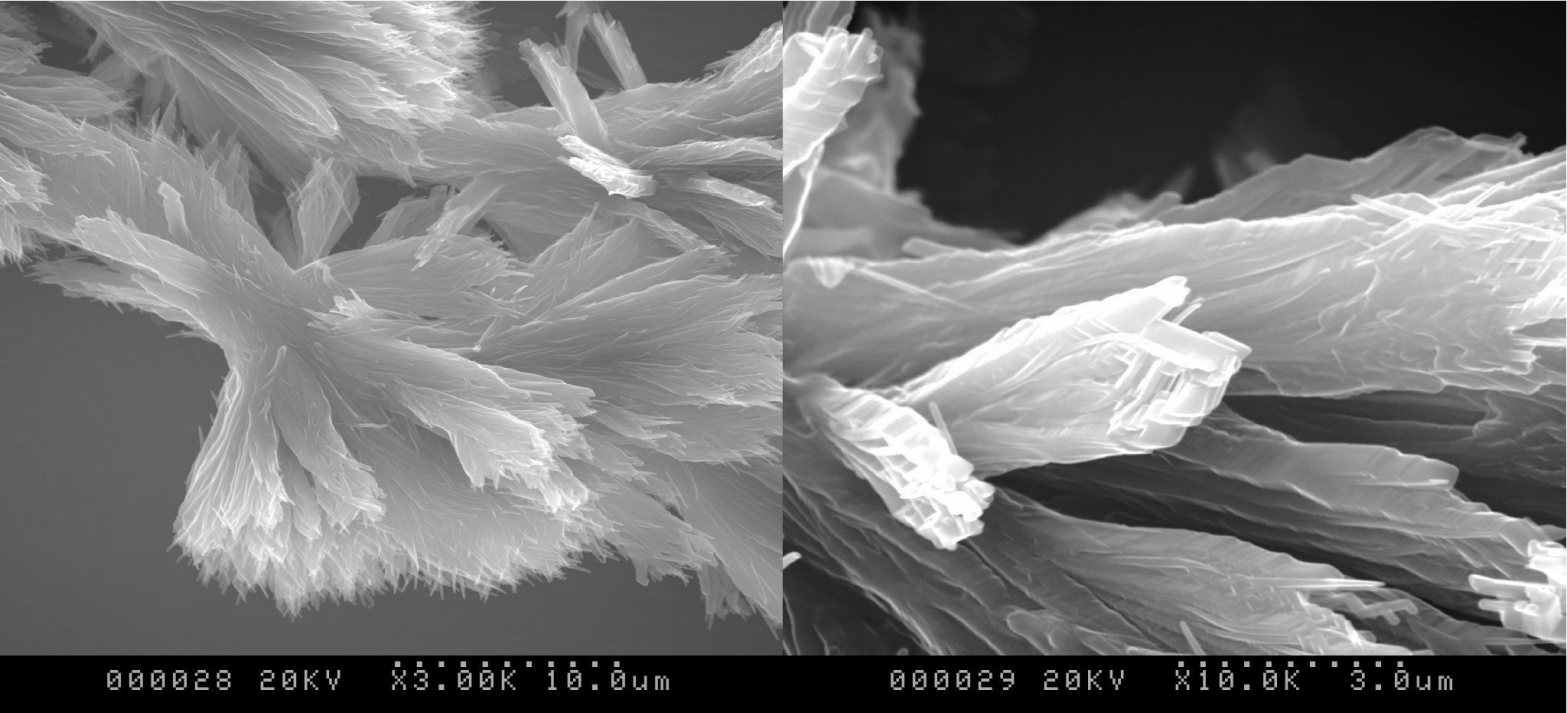
SEM micrographs of β-Ala-His-EO_2_-C_14_ hydrogels after drying.

The structure of the gel was studied by simultaneous small and wide-angle X-ray scattering (SAXS and WAXS) measurements (Figure 3). At low concentrations of the gelator (2% w/v) only the scattering profile was observed. However, when the concentration of the gelating compound was increased to 50% w/v, a broad diffraction peak appeared in the low-angle region suggesting the presence of a poorly ordered micellar phase with a periodicity distance of 6.9 nm. The observed d-spacing was slightly larger than twice the fully extended length of a single surfactant molecule, 2 × 3.4 nm (calculated using MOPAC method, CS Chem Office). Upon heating, the intensity of the peak decreased drastically which can be attributed to a phase transition between 50 and 60 °C. The phase transition temperature was in agreement with the one observed by the dropping ball method at the same concentration, and was higher than the sol-to-gel transition temperature of the 2% w/v sample. On drying, the fibres aggregate to form larger fibre bundles as shown in Figure 4.

The morphology of the gel was observed by scanning electron microscopy (SEM, Figure 4). The micrographs show bundles tens of micrometers long and more than 1 μm wide containing thin fibres (less than 300 nm). Upon gelation, it appears that self-assembly of the 3D fibres of these low molecular weight molecules results in the formation of fibrous networks.

**Figure 5:**
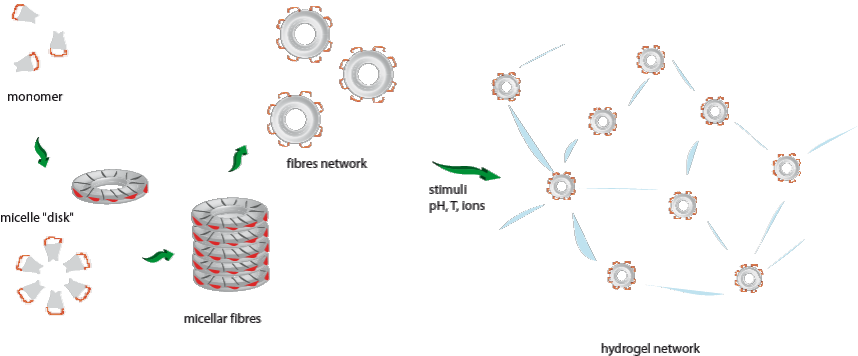
2D/3D self assembling of β-Ala-His-EO_2_-C_14_.

All these results appear to suggest the following gelation mechanism. On reducing the temperature to a certain value, amphiphilic molecules start to interact with each other by H-bonding to form micellar networks. The hydrophobicity of the 1D system is proportional to its length. Once the micellar fibre is too long, and thus too hydrophobic to be solubilised by water, it tends to interact with others fibres to produce 2D/3D networks (Figure 5).

Due to their complexing properties, these compounds are suitable candidates for selective binding of biologically relevant cations such as Cu^2+^ or Ni^2+^, and therefore they offer a straightforward approach for the design of bioactive formulations, and, in particular, for oxidation stress problems. Moreover, these chiral hydrogels could potentially serve as general matrices to host various chiral compounds, either hydrophobic or hydrophilic, and could be used as an enantiomeric sensor or as a separation device for a range of chiral molecules.
